# Comparison of two interferon gamma release assays in the diagnosis of *Mycobacterium tuberculosis *infection and disease in The Gambia

**DOI:** 10.1186/1471-2334-7-122

**Published:** 2007-10-25

**Authors:** Ifedayo MO Adetifa, Moses D Lugos, Abdulrahman Hammond, David Jeffries, Simon Donkor, Richard A Adegbola, Philip C Hill

**Affiliations:** 1 Bacterial Diseases Programme, Medical Research Council Laboratories, Fajara, The Gambia

## Abstract

**Background:**

IFN-γ Release Assays (IGRAs) have been licensed for the diagnosis of latent *Mycobacterium tuberculosis *infection (LTBI). Their performance may depend on assay format and may vary across populations and settings. We compared the diagnostic performance of an in-house T -cell and commercial whole blood-based IGRAs for the diagnosis of LTBI and TB disease in The Gambia.

**Methods:**

Newly diagnosed sputum smear positive cases and their household contacts were recruited. Cases and contacts were bled for IGRA and contacts had a Mantoux skin test. We assessed agreement and discordance between the tests and categorized a contact's level of *M. tuberculosis *exposure according to where s/he slept relative to a case: the same room, same house or a different house. We assessed the relationship between exposure and test results by multiple logistic regression.

**Results:**

In 80 newly diagnosed TB cases, the sensitivity of ELISPOT was 78.7% and for QFT-GIT was 64.0% (p = 0.047). Of 194 household contacts 57.1% and 58.8% were positive for ELISPOT and QFT-GIT respectively. The overall agreement between both IGRAs for LTBI in contacts was 71.4% and there was no significant discordance (p = 0.29). There was significant discordance between the IGRAs and TST. Neither IGRA nor TST had evidence of false positive results because of Bacille Calmette Guérin (BCG) vaccination. However, agreement between QFT-GIT and TST as well as discordance between both IGRAs and TST were associated with BCG vaccination. Both IGRAs responded to the *M. tuberculosis *exposure gradient and were positively associated with increasing TST induration (p = 0.003 for ELISPOT and p = 0.001 for QFT-GIT).

**Conclusion:**

The ELISPOT test is more sensitive than the QFT-GIT for diagnosing TB disease. The two tests perform similarly in the diagnosis of LTBI in TB contacts. Significant discordance between the two IGRAs and between each and the TST remain largely unexplained.

## Background

An estimated one third of the world's population is infected with *Mycobacterium tuberculosis *[1]. Interventions against latent *M. tuberculosis *infection (LTBI) may have a key role in controlling the epidemic [2]. Until recently, the identification of persons with LTBI was only possible using the tuberculin skin test (TST). However, the TST is subject to confounding by prior Bacille Calmette Guérin (BCG) vaccination in certain settings, boosting, reader variability, and false negative results in immunosuppressed persons in particular [3-5].

T-cell interferon gamma (IFN-γ) release assays (IGRAs) are now licensed for the diagnosis of LTBI [6-8]. They measure IFN-γ production by sensitized T-cells in response to stimulation by relatively specific *M. tuberculosis *antigens. IGRAs differ from each other mainly with respect to the technique of IFN-γ detection (enzyme linked immunospot; ELISPOT vs. enzyme linked immunosorbent assay; ELISA) and the samples utilized (peripheral blood mononuclear cells vs. whole blood). QuantiFERON-TB Gold in-tube (QFT-GIT, Cellestis Limited, Carnegie, Australia) has some advantages over ELISPOT: samples can be stored and run in batches, require less investment in equipment, and the assay is technically easier to perform. QFT-GIT uses overlapping peptides of ESAT-6, CFP-10, and TB 7.7 (from Rv2654) antigens. Antigenic stimulation occurs within the tube used to collect the blood sample.

The performance of IGRAs may vary across populations, in relation to background disease prevalence, prevalence of HIV infection, malnutrition, BCG vaccination, exposure to non-tuberculous mycobacteria and other factors [6-8]. In this study we compared the diagnostic performance of an ex vivo ELISPOT and the QFT-GIT for the diagnosis of LTBI and disease in a TB-endemic tropical setting.

## Methods

### Participants

As part of an ongoing case contact study, index cases ≥15 years old with pulmonary tuberculosis (PTB) were recruited between September 2004 and February 2006, from the major government health centre of the Greater Banjul area and the Medical Research Council Laboratories outpatients' clinic. Included cases had two sputum samples positive for acid-fast bacilli by Ziehl-Neelson stain and *M. tuberculosis *on culture. After appropriate counselling, they had blood sampled for HIV test and for IGRAs.

Household contacts were visited, invited to give informed consent and interviewed. They were included if they were ≥15 years of age (the age restriction of the overarching study) and lived the majority of the time on the same compound as a case. They were not eligible if treated for TB in the past year and were excluded if diagnosed with TB within 1 month of recruitment. Contacts underwent a PPD skin test (2 TU, PPD RT23, Statens Serum Institut, Copenhagen, Denmark) using the Mantoux technique and induration was recorded at 48–72 hours. Those with a positive skin test (mean induration diameter ≥10 mm) were offered a chest X-ray and those with symptoms underwent a clinical assessment. Those with TB disease were referred to the National TB Control Programme for free treatment. There is no current practice of preventive treatment in The Gambia.

Four weeks after having a Mantoux test, household contacts that were selected for this comparison had samples taken for ELISPOT assay, QuantiFERON-TB Gold In-Tube (QFT-GIT) assay and HIV test. The overarching case contact study has, as per protocol, blood sampling 1 month after skin test. Contacts were selected for IGRA testing using an even consecutive balanced sampling frame that allowed for the possibility that more individuals who were skin test positive might choose to take part than those who were skin test negative. Fresh samples from all participants were processed onsite.

The study was approved by the joint Gambia Government/MRC Ethics Committee.

### Laboratory procedures

Sputum smears were prepared and stained with auramine-phenol[9] and confirmed by Ziehl-Neelson (Z-N). Decontaminated specimens were inoculated into one slope each of Lowenstein-Jensen medium (L-J) containing glycerol and sodium pyruvate respectively and one vial of BACTEC 9000 MB media for isolation of *M. tuberculosis*. All mycobacterial cultures were identified and confirmed using standard procedures as described previously [10].

The *ex-vivo *ELISPOT assays for IFN-γ were performed as previously described [11]. For this study, synthetic, sequential peptides spanning the length of ESAT-6 and CFP-10 (ABC, Imperial College, London, UK) were used. Each peptide was 15 amino acids long and overlapped its adjacent peptide by 10 residues. ESAT-6 and CFP-10 peptide pools were used at 5 μg/ml. The positive control was Phytohaemaglutinin (PHA 5 μg/ml; Sigma-Aldrich, UK). All antigens were tested in duplicate wells. Assays were counted with an automated ELISPOT reader (AID-GmbH, Strasberg, Germany). The spot forming unit (SFU) numbers counted in each well were automatically entered into a database. Supplementary details were added by double data entry. Positive test wells were pre-defined as containing at least 8 SFUs/well/2 × 10^5 ^PBMCs more than negative control wells [12]. For a positive ESAT-6/CFP-10 result it was necessary for one or more pools of overlapping peptides to be positive. PHA wells were set to at least 150 SFUs/well/2 × 10^5 ^PBMCs above negative control wells. Negative control wells were required to have less than 20 SFUs/well/2 × 10^5 ^PBMCs [12]. The ELISPOT was considered to have failed if the specifications for the negative control and PHA wells were not met.

The QuantiFERON^®^-TB Gold In-Tube (QFT-GIT) assay was carried out according to the manufacturer's instructions[13] In the first step of this test, 1 ml of whole blood was collected directly into 2 × 1 ml blood collection tubes containing either TB-specific antigens (ESAT-6, CFP-10, and TB7.7) or nil (negative control). They were incubated at 37°C for 16 to 24 h before plasma was harvested and stored at -20°C until determination of IFN-γ levels by QFT-GIT ELISA using a recombinant human IFN-γ standard. The IFN-γ levels were measured in international units (IU) with a Dynex ImmunoAssay System ELISA reader version 6.0 (Dynex Technologies, West Sussex, UK). The raw data were entered into QFT-GIT analysis software. A positive result was defined as IFN-g concentration in antigen stimulated tube minus that in the negative control tube ≥0.35 IU/mL. There was no positive control tube.

Testing for HIV-1 or HIV-2 infection was by competitive enzyme linked immunosorbent assays (Wellcome Laboratories, Kent, UK) and Western blot (Diagnostics Pasteur, Marnes-la-Coquette, France).

### Ascertainment of exposure

TB contacts were categorized according to where they slept: in the same bedroom as the index case, a different bedroom in the same house, or in a different house in the same compound [14,15].

### Data management and analysis

All data were entered using double data entry into an ACCESS database and checked for errors. The concordance between the ELISPOT, QFT-GIT and TST was assessed by calculation of a kappa statistic and discordance by McNemar's test. A random effects logistic regression model, taking into account household clustering, was used to assess the relationship between sleeping proximity to an index case and test results. Age and sex were included in the analysis at the outset. Other variables assessed for inclusion in the model were ethnicity, BCG scar status, smear grade and duration of cough of the respective index case. The likelihood ratio test was used to test for trend and for interaction between variables. All statistical analyses were conducted using Stata software (version 9; Stata Corp, College Station, TX).

## Results

Three hundred and twenty individuals were recruited for the overarching study, 194 household contacts and 80 TB cases were selected for this comparison study. Just over 60% of the household contacts were female while 60% of cases were male. Only 10 (3.7%) of those tested overall were HIV positive (Table 1). One hundred and eighty-seven (96.4%) had chest X-rays done at recruitment: six (3.6%) had some radiological abnormalities present. All 6 were asymptomatic. One had been treated for TB in the past; and 2 were diagnosed with TB disease over the next few months. The results for the 2 cases were not included in those for the 80 TB cases that were sampled at recruitment.

**Table 1:** Characteristics of study population

Characteristics	Contacts Sleeping proximity gradient			Cases	ALL
			
	Separate house	Separate room	Same room		
	n = 38	n = 115	n = 41	n = 80	n = 274
Age, years					
Mean	29.7	30.0	34.3	31.2	31.0
Median (IQR)	26.5(19–36)	25.0(18–36)	32.0(25–44)	30(23–36)	28(20–37)
Sex					
Male	18(47.4)	38(33.1)	20(48.8)	51(63.8)	127(46.4)
Female	20(52.6)	77(66.9)	21(51.2)	29(36.2)	147(53.6)
Ethnic group					
Madinka	11(28.9)	47(40.9)	17(41.5)	26(32.5)	101(36.9)
Jola	7(18.4)	32(27.8)	7(17.0)	19(23.8)	65(23.7)
Wollof	2(5.3)	10(8.7)	5(12.2)	8(10.0)	25(9.1)
Fula	8(21.1)	7(6.1)	4(9.8)	11(13.7)	30(11.0)
Others	10(26.3)	19(16.5)	8(19.5)	16(20.0)	53(19.3)
Clinical Findings					
BCG scar					
Absent	20(55.6)	48(43.6)	24(66.7)	34(42.5)	126(46.0)
Present	16(44.4)	59(53.7)	9(25.0)	19(23.8)	103(37.6)
Uncertain	0(0.0)	3(2.7)	3(8.3)	27(33.7)	45(16.4)
HIV results*					
Positive	0(0.0)	3(2.6)	0(0.0)	7(8.8)	10(3.7)

Data are no. (%) of persons unless stated otherwise.

BCG, Bacille Calmette-Guérin

*Data are for 178 contacts and 77 cases.

**Figure 1:**
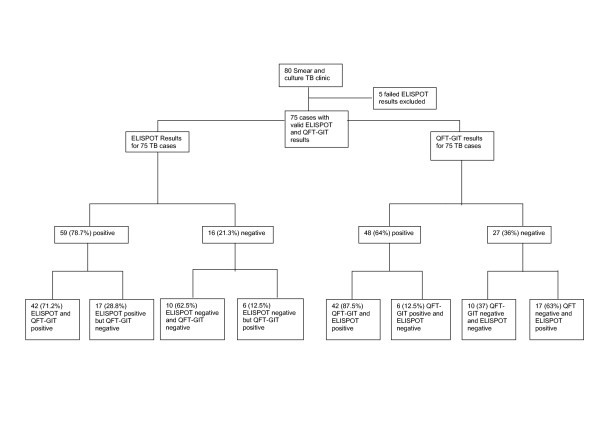
Distribution of ELISPOT and QFT-GIT results in TB cases.

All 80 smear positive TB index cases had both ELISPOT and QFT-GIT tests performed. Figure 1 shows the distribution of the IGRA results in index cases. None of the QFT-GIT tests in cases failed; while there were 5 (6.25%) failed ELISPOT tests (positive control criteria not met). Of the 75 cases that had both ELISPOT and QFT-GIT results, 59 (78.7%; 95% CI: 67.7–87.2%) cases had a positive ELISPOT while 48 (64.0%, 95%CI: 51–73%) were positive by QFT-GIT. This difference in sensitivity was statistically significant (p = 0.047). While there was significant discordance (McNemar's test: p = 0.02) between the two tests as shown in figure 1, the overall agreement in TB cases was 69.3% (concordance, κ = 0.27).

Valid ELISPOT and QFT-GIT results were available for 182 and 187 contacts respectively. The difference in the proportion of failed tests was not significant (p = 0.24). The 7 QFT-GIT results excluded all failed the criteria for negative controls as well as 5 (42%) of the excluded 12 ELISPOT results – the other 7 (58.3%) did not meet the criteria for the positive control. Overall, 104 (57.1%) of 182 contacts tested positive to the ELISPOT (95% CI, 49.9%–64.4%) and 110 (58.8%) of 187 were QFT-GIT positive (95% CI, 51.7%–65.9%). A total of 175 had both ELISPOT and QFT-GIT results. Twenty-six (25.5%) QFT-GIT positive contacts were ELISPOT negative while 24 (24%) ELISPOT positive contacts were QFT-GIT negative. The overall agreement between the two tests was 71.4% (κ = 0.41; 95%CI, 0.27–0.56) and there was no significant discordance (p = 0.29).

**Figure 2:**
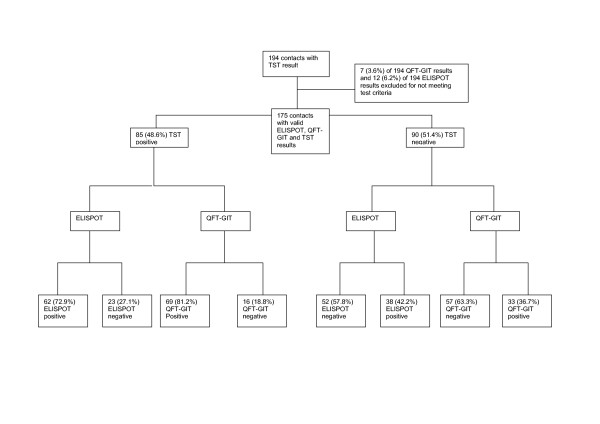
Distribution of ELISPOT, QFT-GIT and TST results in household contacts.

Figure 2 shows the distribution of IGRA results by TST in household contacts. Just under half were skin test positive (48.6%). Twenty-three (27.3%) TST positive contacts were ELISPOT negative and 38 (42.2%) ELISPOT positive contacts were TST negative. The overall agreement between the ELISPOT test and TST was 65% (κ = 0.31; 95% CI: 0.16–0.44, discordance, p = 0.055). Sixteen (18.8%) TST positive contacts were QFT-GIT negative and 33 (36.2%) QFT-GIT positive contacts were TST negative. The overall agreement between QFT-GIT and TST was 71.1% (κ = 0.43; 95% CI, 0.29–0.57) and there was significant discordance (p = 0.007).

A BCG scar was clearly visible in 84 (43.3 %) household contacts. The univariate odds of a positive ELISPOT or QFT-GIT test in those BCG scar positive compared to those without scars was1.23 [0.63–2.4; p = 0.54] and 0.92 [0.41–2.1; p = 0.84] respectively, both remaining non-significant after adjustment for possible confounding variables. The agreement between ELISPOT and TST did not differ significantly according to BCG scar status being 69.6% (κ = 0.38; 95%CI: 0.16–0.60, discordance p = 0.4) in those contacts with a BCG scar and 62.4% (κ = 0.26; 95% CI: 0.06–0.47, discordance p = 0.03) in those without a BCG scar. In contrast, agreement between QFT-GIT and TST was significantly different by BCG scar status (p = 0.03), being 76.5%, (κ = 0.52; 95%CI: 0.31–0.73, discordance p = 0.11) in those with a scar and 68.2% (κ = 0.37; 95%CI: 0.17–0.58, discordance p = 0.02) in those without a scar.

**Figure 3:**
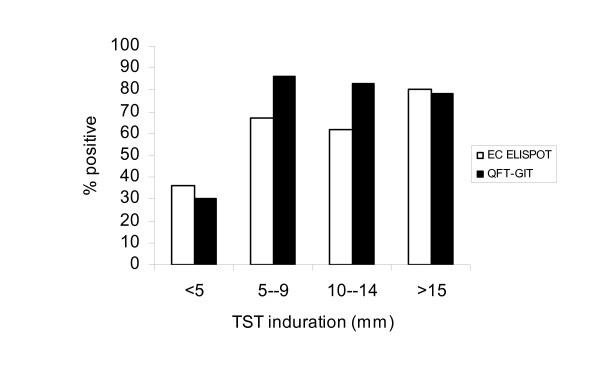
Proportion of positive EC ELISPOT and QFT-GIT across TST categories.

In contacts, when a positive TST was defined as ≥5 mm, 86 (81.1%; 95%CI: 73.7–88.6%) of 106 TST positives were also QFT-GIT positive compared to 73 (71.4%; 95%CI: 62.8–80.3%) of 102 for the ELISPOT but this difference in proportions was not significant (p = 0.10). With a positive TST defined as ≥10 mm, 73 (81.1%; 95%CI: 73.0–89.2%) of 90 were QFT-GIT positive and 72 (72.7%; 95%CI: 63.4–82.0%) of 88 had positive ELISPOTs. When a definition of ≥15 mm was used, 36 (78.3%; 95%CI: 66.3–90.2%) of 46 had positive QFT-GIT, similar to the ELISPOT with 37 (78.7%; 95%CI: 67.0–90.4%) of 47. These differences in proportions were also not significant (p = 0.18 and p = 0.96 respectively). Figure 3 shows the proportions of contacts positive by ELISPOT and QFT-GIT across respective TST categories. There was an association between both ELISPOT and QFT-GIT results and TST categories (χ^2 ^= 21.6, p < 0.001 and χ^2 ^= 50.6, p < 0.001 respectively). With increasing TST induration there was a trend towards increase in the proportions of contacts with positive ELISPOT and QFT-GIT results and this was statistically significant (p = 0.003 and p = 0.0001 respectively).

The odds of test positivity were also not related to sputum smear grade (OR = 0.8 [0.3–1.4] for ELISPOT; QFT-GIT OR = 0.5 [0.1–1.6), or duration of cough (OR, 1.0 [0.9–1.1] for both tests). The univariate odds of a positive result across the *M. tuberculosis *exposure categories (represented as sleeping proximity to a case) are shown in Table 2. While neither showed as dramatic a change in positivity across the exposure gradient as the skin test, both IGRA tests responded significantly to increasing exposure to an index case as shown in table 2. However, significance was lost for both tests after adjusting for age, sex and ethnicity.

**Table 2:** Univariable and multivariable odds ratios determined by logistic regression (household as random effect), for sleeping proximity as a surrogate marker of exposure to *M. tuberculosis*

	ESAT-6/CFP-10 ELISPOT (n = 182)				Quantiferon (n = 187)				TST (n = 194)........			
			
	Positive results No.(%) of contacts	Unadjusted OR (95% CI)	Adjusted OR (95% CI)	p-value	Positive results No. (%) of contacts	Unadjusted OR (95% CI)	Adjusted OR(95% CI)	p-value	Positive results No.(%) of contacts	Unadjusted OR(95% CI)	Adjusted OR(95% CI)	p-value
**Sleep proximity**												
Different house	17(48.6)	1	1		18(50.0)	1	1		38 (34.2)	1	1	
Different room	63(57.8)	1.8(0.6–5.1)	2.3(0.7–7.0)		64(57.7)	1.4(0.6–3.1)	1.5(0.6–3.6)		55 (47.8)	1.8(0.8–4.1)	2.4(0.9–6.5)	
Same room	24(63.2)	2.3(0.6–8.5)	4.2(1.0–18.0)	0.15**	28(70.0)	2.6(0.9–7.6)	3.8(1.2–12.5)	0.08**	25 (61.0)	3.2(1.2–8.9)	4.8(1.3–17.1)	0.06**

*Positive result defined as a mean induration diameter of ≥10 mm.

**p values for linear trend,

## Discussion

This study now provides a comparison of an ELISPOT test with QFT-GIT for the diagnosis of *M. tuberculosis *infection and disease in a resource poor TB-endemic tropical setting. Our results suggest both IGRAs, the QFT-GIT and our in house ELISPOT perform similarly in the diagnosis LTBI in contacts after recent exposure to a known TB case. However, the ELISPOT is more sensitive in diagnosing active PTB in cases.

The sensitivity of 78.7% for the ELISPOT is within our previously published range 72.6–89.8% [16] and 77–97% reported elsewhere in literature [7]. Estimates of 55–88% have been obtained from studies of QFT-GIT among individuals with active TB – our figure of 64.0% is within this reported range [17-23]. There are other reports of comparable sensitivity for both IGRA test formats in cases [24,25]. It is possible that the lack of a positive control tube for QFT-GIT has led to false negative results. The manufacturers have since introduced a positive control tube which we now use in our new QFT studies.

This study has taken advantage of a reproducible gradient of exposure to *M. tuberculosis *in The Gambia. Both tests responded appropriately to a gradient of recent exposure to *M. tuberculosis *and there was no significant divergence in results according to level of exposure. While we do not find strong evidence for sensitivity difference in case contacts, the tentative consensus from published literature is that ELISPOT does have better sensitivity with respect to LTBI [7].

Consistent with other studies, we show considerable discordance between the TST and the IGRAs and between the IGRAs themselves. Our results show significant discordance between the IGRAs in cases. Although about a quarter of ELISPOT or QFT-GIT positive contacts were negative with the other test, the discordance was not statistically significant. Discordance between IGRAs in cases and contacts has been described in several reports [18,21,26]. Similar to our results, more of the reported discordance is due to QFT-GIT negative and ELISPOT positive combinations. Some discordance may be related to test formats and inclusion of an additional antigen in QFT, but unfortunately remains poorly understood. Reported discordance between each IGRA and the TST ranges from 10–40% and is most often due to negative TST and positive IGRA results [6,7]. Discordance between IGRAs and TST has been related to BCG vaccination, [27-29] and to superior specificity of the IGRAs, at least in certain settings[15]. More recently, different rates of test conversion and reversion over time have been noted [30,31].

The absence of any significant effect of BCG vaccine on the TST, ELISPOT and QFT-GIT has remained a constant feature in Gambian studies, [15,32] and has been reported in other tropical settings, where BCG is given at birth [33-35]. It is inconsistent with a published systematic review and meta analysis which reported increased likelihood of a positive TST in BCG vaccinated persons [36]. A weakness of our study, and most others, is reliance on the presence or absence of a BCG scar because of the difficulties in obtaining accurate immunization histories and/or records.

That the TST was not done at the same time as the IGRAs may also be a considered a weakness of this study, as it makes any comparison of IGRA with the TST, subject to mis-classification bias. However, our primary goal was not to compare the IGRAs with the TST but to evaluate the IGRAs in relation to each other. The TST is subject to boosting. Although, there are some reports of possible boosting, by a previous TST, of the QFT-GIT and not the ELISPOT, large conclusive studies are lacking [37-39]. The 1-month time-lag for the overarching study was put in place to minimise any transient boosting effect on T cell responses. However, it is possible these results could have been affected by test conversion, reversion and boosting.

The higher test failure rate of the ELISPOT, compared to the QFT-GIT, in our study is inconsistent with other studies, where the opposite has been reported [18,21]. We note that we have more stringent criteria for an acceptable test result for ELISPOT than the commercial ELISPOT based test [40]. The spot counting criteria we used are based on our published study where we used mathematical tools to identify a cut-off [12]. Furthermore, the lack of a positive control for QFT-GIT, as mentioned above, may have been a factor.

A limitation of IGRAs is their relatively high cost and need for laboratory infrastructure. The greater through-put of samples for QFT-GIT gives it some advantage in this light. However, one would need to demonstrate significant further advantage over the TST than is seen here, to justify the investment for developing countries in particular. The outstanding question to resolve now is whether IGRAs show improved predictive value for progression to active disease, once an individual is infected with *M. tuberculosis*. This will require large, possibly multi-site, follow-up studies of TB case contacts and molecular subtyping of case and secondary case isolates. Such follow-up studies are underway in The Gambia.

## Conclusion

This study has shown that an in-house ELISPOT has greater sensitivity than the QFT-GIT for diagnosing active TB disease, and perform similarly with respect to *M. tuberculosis *infection in The Gambia.

## Competing interests

The author(s) declare that they have no competing interests.

## Authors' contributions

IMO contributed to study design, conducted field and clinical activities, and was involved interpretation of laboratory data and data analysis.

PC conceived of the study, and participated in its design and coordination, and was involved in data analysis.

DJ contributed to design of the study and data analysis.

SD contributed to study design and managed the data generated.

ML carried out ELISPOTs and was involved in data interpretation.

AH carried out QFT-GIT assays and was involved in data interpretation.

RA provided overall supervision and was responsible for TB diagnostics

All authors read and approved the final manuscript.

## Financial support

This study was funded by the Medical Research Council (United Kingdom), QFT-GIT kits were provided free by Cellestis Limited, Carnegie, Australia. IMOA receives financial support from the European and Developing Countries' Clinical Trials Partnership (EDCTP).

## Pre-publication history

The pre-publication history for this paper can be accessed here:


